# Variable habitat conditions drive species covariation in the human microbiota

**DOI:** 10.1371/journal.pcbi.1005435

**Published:** 2017-04-27

**Authors:** Charles K. Fisher, Thierry Mora, Aleksandra M. Walczak

**Affiliations:** 1 Laboratoire de physique théorique, CNRS, UPMC and École normale supérieure, Paris, France; 2 Laboratoire de physique statistique, CNRS, UPMC and École normale supérieure, Paris, France; Johns Hopkins Bloomberg School of Public Health, UNITED STATES

## Abstract

Two species with similar resource requirements respond in a characteristic way to variations in their habitat—their abundances rise and fall in concert. We use this idea to learn how bacterial populations in the microbiota respond to habitat conditions that vary from person-to-person across the human population. Our mathematical framework shows that habitat fluctuations are sufficient for explaining intra-bodysite correlations in relative species abundances from the Human Microbiome Project. We explicitly show that the relative abundances of closely related species are positively correlated and can be predicted from taxonomic relationships. We identify a small set of functional pathways related to metabolism and maintenance of the cell wall that form the basis of a common resource sharing niche space of the human microbiota.

## Author summary

The human body is inhabited by a vast number of microorganisms comprising the human microbiota. The species composition of the microbiota varies considerably from person-to-person and the relative abundances of some species rise and fall in concert. We introduce a mathematical model where differences in habitat conditions cause most of the variability of the microbiota. A statistical analysis shows that variable habitat conditions are sufficient for explaining the patterns of variation observed across a healthy human population and, as a result, the correlation between the relative abundances of two species reflects how closely related they are rather than how they directly interact with each other.

## Introduction

Species in an ecosystem interact with each other and with their environment. Both types of interactions leave an imprint on the composition and diversity of a community. Two species competing for exactly the same resources engage in a struggle for existence [1]. In the end, one species will win the competition by driving the other to extinction. As a result, one might expect that closely related species rarely occupy the same habitat where they would risk being drawn into competition. On the other hand, species that survive in the same habitat must share many common features [2]. Thus, the rise and fall of a common resource may cause the abundances of similar species to rise and fall in concert. These opposing ecological forces simultaneously push and pull on species abundances to shape the composition of a community.

Ecological processes operate on the thousands of microbial species that inhabit the human body [3–6] just as they operate on the Amazon rainforest. Technological advances have recently made it possible to study the human microbiota using 16S ribosomal RNA tag-sequencing and whole genome ‘shotgun’ metagenomics [7]. Variability in the composition of the microbiota can be studied in two ways. Longitudinal studies follow the relative abundances of the species in a single bodysite of a particular person over time [8]. Cross-sectional studies examine the relative abundances of the species in a single bodysite across a sample of many different people [9]. These studies have demonstrated that the composition of the human microbiota exhibits three qualitative scales of variation [10, 11]: there are small-scale fluctuations in relative species abundances through time, there are medium-scale variations in species composition from person-to-person, and there are large-scale differences in species composition between different bodysites.

**Fig 1:**
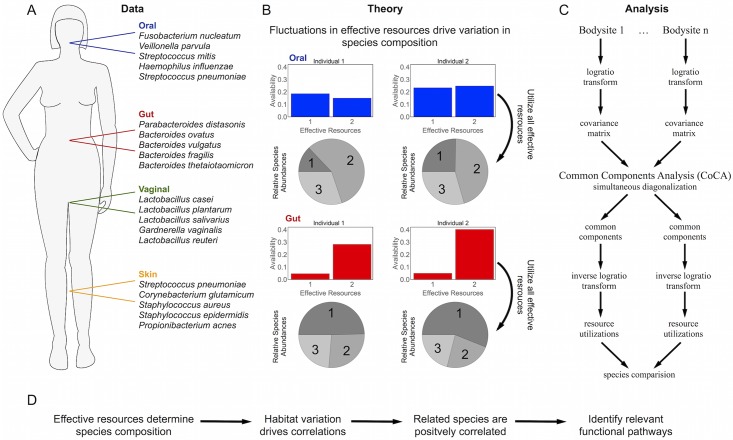
Schematic outline of the paper. A) Data on the microbial species composition of the gut, oral cavity, vagina, and skin from the Human Microbiome Project were obtained from MG-RAST. One hundred highly abundant species were selected for further study (S1 Text). The top five most abundant species in each bodysite are shown. B) We propose a theory for the composition of the microbiota based on a Maximum Diversity Hypothesis, which says that the equilibrium relative species abundances in a community maximize the diversity of the community while ensuring that all niches are fully utilized. As a consequence, variability from person-to-person in the species composition within a bodysite results from variability in the availabilities of some ‘effective resources’ that define the space of niches. C) An overview of the steps in the Common Components Analysis (CoCA) algorithm. Covariance matrices are computed from the log-ratio transformed relative abundances in each bodysite. The covariance matrices are simultaneously diagonalized to obtain a basis of common components, and an inverse log-ratio transform is applied obtain the inferred effective resource utilizations. D. Overall logical flow of the paper. Characterizing the effective resources allows us to identify the functional pathways which must be conserved to utilize a common habitat.

In this work we quantitatively explore the idea that variations in species abundances between different sites can be explained mainly through the local variations in resource availability. Concretely, we reanalyze data from the Human Microbiome Project (HMP) [3–5, 12] on the species composition of different bodysites (i.e., gut, skin, vagina, and oral cavity; Fig 1). To develop the analysis method, we start from a theoretical model that assumes maximal diversity of species and derives a relationship between species abundance and resource availability. We then use the results of this model to guide the joint analysis of the datasets from different body sites in the HMP project, introducing a new Common Component Analysis (CoCA) method. The general idea is that if the same species exist in different body sites, the same resources must also be present at these body sites. We find that this intuition is correct by showing that the covariance of species abundances at specific body sites can be simultaneously projected into the same basis that describes the availability of effective resources. This means that the abundances of species at different body sites are driven by the same set of resources, just different resources are of varying importance at different body sites. These results cannot be reproduced from randomized data and reflect an underlying global set of ecological resources shared between body sites.

Our method identifies species that share common resources. To understand the source of this sharing, we look for similarities between the species. We find that species that share common resources are also closely related taxonomically, suggesting that evolution is constrained by ecology. We further back this observation by identifying specific metabolic pathways that are conserved between species identified as close using our resource sharing analysis.

The goal of this work is three-fold. First, we introduce a new analysis technique that identifies covariations among components in different subsets that are driven by the same process (CoCA). Second, by successfully applying the CoCA analysis technique to HMP data, we show that the diversity of the microbiome at different body sites is shaped by common biological processes. Third, in the case of the specific problem of species at different ecological sites, we make the biological point that microbiome species that share the same resources are also closely related taxonomically and we back this fact by identifying shared pathways. The methodological developments are presented in the “Theory” subsection of the Results and the biological results in the“Analysis” subsection. For readers predominantly interested in the biological results the “Analysis” subsection can be read independently of the “Theory” subsection.

In addition to presenting statistical evidence for the observations described above, we formulate a hypothesis (in the form of a mathematical model) that explains their origin. Our model is inspired by MacArthur’s famous model of competition [1, 13–15], but is adapted to account for the compositional nature of metagenomic survey data. We demonstrate that *habitat variability* is sufficient to explain the medium-scale variations in species composition observed in a cross-sectional study of the human microbiota. As a result, the relative abundances of closely related species are positively correlated—they rise and fall in concert as habitat conditions vary from person-to-person. Therefore, cross-sectional studies allow us to extract a wealth of information about the influence of species traits and habitat properties on community composition using advanced statistical techniques.

## Results and discussion

### Theory

#### MacArthur’s consumer-resource model and the definition of effective resources

Before we present the method developed in this paper, for completeness we first briefly discuss a classic deterministic model of resources utilization. In a series of pioneering papers [1, 13, 14], Robert MacArthur developed a theory that explicitly linked the dynamics of resource and consumer species in an ecosystem. This section will provide a brief, non-technical discussion of MacArthur’s theory to provide some context for our work. The consumer-resource model describes the *absolute abundances* of the species in a community where *M* of the species are resources *μ* = 1, …, *M* and *N* are consumers *i* = 1, …, *N*. Assuming that the dynamics of the resource species are much faster than the dynamics of the consumers, the dynamics of the consumer species are governed by a system of generalized Lotka-Volterra equations. MacArthur showed that the equilibrium *absolute abundances* of the consumer species correspond to the point where the consumers make the most effective use of the resources.

Species in MacArthur’s model are characterized by resource utilizations—that is, each consumer species *i* is described by a vector of length *M* containing the rate that species *i* depletes resource *μ* = 1, …, *M*. The resource utilizations form the basis of a vector space that one could call a niche space. The community dynamics cannot have an internal equilibrium (i.e., with all species coexisting) unless the basis is either complete (i.e. *M* = *N*) or overcomplete (i.e. *M* > *N*). It is important to keep in mind, however, that there are many mechanisms that lead to coexistence in real communities where spatial and stochastic effects are important [16]. Although the resource basis was defined in a biologically meaningful way by MacArthur, there is nothing that restricts us to this exact choice. In fact, any set of *N* linearly independent vectors constructed from linear combinations of the resource utilizations will also be an equally valid basis. This leads to an infinite choice of potential bases that have exactly the same dynamics (thus, they will be indistinguishable by a statistical model), with each possible set of basis vectors providing a different view of the niche space. Although these basis vectors are related to the original resource utilizations they are, indeed, different so we have adopted the term “effective resources” to reflect the ambiguity in relating the inferred bases that we will use in this paper to the underlying resources.

#### Adapting resource models to relative abundances with the maximum diversity hypothesis

Our first step to analyze the HMP microbiome dataset is to build a theoretical model for species abundance covariation that will guide our inference. Metagenomic survey experiments typically provide measures of relative, rather than absolute, species abundances. That is, the experiments do not provide an accurate measure of the overall size of the population. Therefore, it is necessary to develop a theory that directly models relative abundances. Our theory is inspired by two aspects of MacArthur’s consumer resource model [1, 13–15]: first, species are described by vectors of resource utilizations that act as a basis of a linear niche space and, secondly, that the equilibrium relative abundances correspond to the point where the consumers make the most effective use of the resources. Specifically, our model is based on an ecological hypothesis we call the maximum diversity hypothesis:

**Maximum Diversity Hypothesis:** The equilibrium relative species abundances in a community maximize the diversity of the community while ensuring that all effective resources are fully utilized.

Many mechanisms that may lead to high diversity communities (e.g. see [16]) have been identified, but we will not attempt a mechanistic derivation of the maximum diversity hypothesis in this work. Instead, we will show that the maximum diversity hypothesis implies that cross-sectional covariances in log-ratio transformed relative abundances can be described by a statistical factor model and that this model is consistent with observations in the human microbiome.

To formalize the model, we suppose that a particular community has *M* effective resources that define the dimensions of a niche space. Note that we use the term ‘effective resources’ in an abstract way that captures all of the abiotic and biotic factors that affect the species composition within a community. Each effective resource *μ* has an availability *V*_*μ*_, which varies between different environments. It is the variation in the availabilities of the effective resources that drives variation in species composition between communities. In a community composed only of species *i*, an amount *V*_*iμ*_ of effective resource *μ* will be utilized. In other words, *V*_*iμ*_ describes the ability of species *i* to utilize effective resource *μ*. Note that *V*_*iμ*_ can be positive, in which case species *i* depletes effective resource *μ*, or it could be negative, in which case species *i* adds more of effective resource *μ* to the environment. For example, a bacterium may secrete a metabolite that is utilized by other species. Finally, we quantify the diversity of a community using the Shannon entropy *H*[***x***] = −∑_*i*_ *x*_*i*_ log *x*_*i*_ [17, 18], where *x*_*i*_ is the abundance of species *i*. Following the maximum diversity hypothesis, the equilibrium relative species abundances can be obtained by maximizing *H*[***x***] subject to constraints *V*_*μ*_ = ∑_*i*_ *V*_*iμ*_*x*_*i*_ and ∑_*i*_ *x*_*i*_ = 1.

We can solve for the equilibrium relative abundances by maximizing the Lagrangian:

$$L\left(x,\lambda ,\gamma \right)=-\sum_{i}x_{i}\log x_{i}+\sum_{\mu }\lambda_{\mu }\left(V_{\mu }-\sum_{i}V_{i\mu }x_{i}\right)+\gamma \left(1-\sum_{i}x_{i}\right)$$ (1)

where *γ* and the λ_*μ*_’s are Lagrange multipliers. The solution is given by

$$\log x_{i}^{*}=\sum_{\mu }\lambda_{\mu }V_{i\mu }+\text{constant}$$ (2)

Eq 2 is of the form:

$$\log\left(\text{species}\;\text{i}\;\text{abundance}\right)=\sum_{\text{resources}}\left(\text{availability}\;\text{of}\;\text{effective}\;\text{resource}\right)\times \left(\text{species}\;\text{i}\;\text{effective}\;\text{resource}\;\text{utilization}\right)+\text{constant}$$

People have different diets, behaviors, and genetic predispositions. Thus, the availabilities of the effective resources λ_*μ*_ vary from person-to-person, causing the relative abundances of the species to vary as well. As a result, the relative abundances of species that use similar effective resources will be correlated, and it is possible to solve an inverse problem to learn the *V*_*iμ*_ (which species use which effective resources) from these correlations (Fig 1C and 1D).

The effective resource utilizations, *V*_*iμ*_, form the basis for the space of log-ratio transformed relative abundances. This space has dimension *N* − 1 because one degree of freedom is lost due to ignorance of the total population size. As a result, the dimension of the niche space inferred from *relative abundances* is, at most, *M* ≤ *N* − 1. Our maximum diversity hypothesis allows for coexistence even if the number of effective resources is much smaller than the number of consumer species, which is a departure from classical theories of consumer-resource systems based on deterministic dynamics, such as MacArthur’s model. Real systems, however, are much more complex than these deterministic models and there exist many well-known mechanisms (e.g. spatial structure, stochasticity [16]) that lead to communities that are more diverse than predicted from classical models. In our statistical inference models used to analyze the microbiome data, we set *M* = *N* − 1 and let the weights of the inferred effective resources determine the dimension of the niche space.

#### Effective resource models are factor models that can be trained with Common Components Analysis (CoCA)

The maximum diversity hypothesis can be implemented as a statistical inference model, which we present on the example of the HMP dataset. The Human Microbiome Project data are grouped into four bodysites (gut, skin, vagina, and oral cavity). There are two standard approaches to analyzing the data in such a situation: first, the data can be lumped together and principal components analysis (PCA) can be performed on all of it, and second, individual PCAs can be performed for each bodysite. The first approach provides a single basis that describes all of the data, allowing one to make direct comparisons of the bodysites within the new basis. However, PCA performed on all of the bodysites at once cannot separate intra-bodysite variability from inter-bodysite differences—it assumes that everything is Gaussian. The second approach focuses only on the intra-bodysite variation, but it provides a different basis for each bodysite making inter-bodysite comparisons difficult. To overcome these limitations, we developed an approach based on the maximum diversity hypothesis, that we call common components analysis (CoCA). The method identifies the single common basis, *V*_*iμ*_, that simultaneously captures the directions of intra-bodysite variability within every bodysite according to Eq 2. Our approach is related to methods called “approximate simultaneous non-orthogonal diagonalization” in the machine learning literature [19–21].

This statistical inference belongs to the class of factor models that perform matrix factorization. Matrix factorization methods are a cornerstone of machine learning and statistics encompassing techniques such as principal components analysis (PCA), principal coordinates analysis (PCoA), archetype analysis, autoencoders, and k-means clustering [22–24]. These methods can be interpreted in two ways: first, they map the data to a new vector space with lower dimension, and second, they represent generative models based on some latent (or hidden) variables. Below, we provide a brief description of the CoCA algorithm.

#### Log-ratio transformations

Before developing a latent factor model for relative species abundances, we need to briefly discuss the log-ratio transformations used to tackle the compositional nature of the data; a more detailed discussion is presented in S1 Text. Compositional data analysis is focused on ratios of relative abundances. In this work, we use an Additive Log-Ratio (ALR) transform that defines the relative abundance of one species as a reference and measures the ratio of every other species to the reference. Distances between ALR transformed relative abundances are not identical to those computed using a natural metric for compositional data (i.e., the Aitchison metric) but they are highly correlated (S1 Fig). In the context of our model, log-ratio transformation means that we cannot estimate the matrix of resource utilizations (**V**) directly. Instead, we can only directly estimate an *N* − 1 × *N* − 1 matrix $\tilde{V}=GV$, where **G** is a matrix that implements the log-ratio transform (Materials and Methods and S1 Text). Before making any statements about the species’ resource utilizations, we have to invert effect of the log-ratio transform to obtain $V=G^{-1}\tilde{V}$ so that our analyses are not sensitive to the particular choice of transformation. We chose the ALR transform in this work because the pseudoinverse **G**^−1^ can be computed efficiently under the assumption that **V** is sparse (Materials and Methods and S1 Text).

#### Diagonalizing the log-ratio transformed covariance matrix

CoCA and PCA have closely related generative models. In each case, a covariance matrix of log-ratio transformed relative abundances takes the form $\Psi =\tilde{V}\Sigma {\tilde{V}}^{T}$ where **Σ** is a diagonal matrix. The differences are as follows. In PCA, **Ψ** is the covariance matrix of the log-ratio transformed relative abundances taken without regard to bodysite and $\tilde{V}$ is assumed to be orthogonal. In CoCA, by contrast, there is an equation for each bodysite s: $\Psi_{s}=\tilde{V}\Sigma_{s}{\tilde{V}}^{T}$. While the diagonal matrix **Σ**_***s***_ is specific to each bodysite, the matrix $\tilde{V}$ is shared across all bodysites, but is not assumed to be orthogonal.

PCoA is a commonly used variant of PCA that performs the factorization on an *implied* covariance matrix derived from a matrix of squared distances. Given that PCA and PCoA are basically the same method (applied in different vector spaces), we will refer only to PCA from now on.

#### CoCA for relative species abundances

As above, let *x* denote an *N* dimensional vector of relative species abundances and ***y*** = **G** log ***x*** denote the (*N* − 1) dimensional vector obtained by taking the additive log-ratio transform of ***x*** (S1 Text). The isometric log-ratio transform could also be have been used for this purpose, but the centered log-ratio transform should not be used because the resulting covariance matrix will not be invertible. Let ${\bar{y}}_{s}$ denote the mean of *y* from bodysite *s*, and **Ψ**_***s***_ its covariance matrix. We search for a single matrix $\tilde{V}$ so that $\Psi_{s}=\tilde{V}\Sigma_{s}{\tilde{V}}^{T}$ is satisfied for all bodysites *s*. We require **Σ**_***s***_ to be diagonal, as in PCA, but do not require $\tilde{V}$ to be orthogonal. This performs a simultaneous diagonalization of the covariance matrices from each bodysite. *It is crucial to remember that a set of unrelated covariance matrices cannot be simultaneously diagonalized (see* S2 Fig Thus, CoCA is not a general analysis technique like PCA; instead, it is only applicable to situations where multiple covariance matrices arise from the same underlying generative process.

Like in PCA, we can formulate CoCA as a generative model, which closely follows the model of maximum diversity of Eq 2. In each bodysite *s*, the log-ratio transformed abundances are generated randomly as

$$y=\tilde{V}\lambda_{s},\;\lambda_{s}={\bar{\lambda }}_{s}+\delta \lambda_{s},$$ (3)

where the mean ${\bar{y}}_{s}=\tilde{V}{\bar{\lambda }}_{s}$ is expressed in the basis of $\tilde{V}$, and *δ***λ**_***s***_ is a Gaussian random vector of zero mean and diagonal covariance matrix **Σ**_***s***_. In this equation, we have assumed that the experimental errors are small relative to the intra-bodysite variation in the relative abundances so that they can be neglected. Maximizing the log-likelihood is equivalent to minimizing the KL-divergence between the assumed distribution and the empirical distribution. The fraction of samples coming from bodysite *s* is *p*_*s*_, and the bodysite labels are known. Therefore, the total negative log-likelihood is a weighted sum of each of the individual negative log-likelihoods. The matrices $\tilde{V}$ and ${\left\{\Sigma_{s}\right\}}_{s=1}^{S}$ can be inferred by minimizing this conditional negative log-likelihood:

$$L\left(\tilde{V},{\left\{\Sigma_{s}\right\}}_{s=1}^{S}\right)=\sum_{s}p_{s}(\text{Tr}\left[\Psi_{s}{\left(\tilde{V}\Sigma_{s}{\tilde{V}}^{T}\right)}^{-1}\right]-\log|{\left(\tilde{V}\Sigma_{s}{\tilde{V}}^{T}\right)}^{-1}\left|\right)$$ (4)

The objective function was minimized with respect to $\tilde{V}$ and the diagonal matrices **Σ**_***s***_ using gradient descent (S1 Text).

#### Inverting the log-ratio transform

The procedure above infers the transformed matrices $\tilde{V}=GV$. In order to interpret these numbers, we first need to invert the log-ratio transform and recover the matrix of resource utilizations in the original space, “$V=G^{-1}\tilde{V}$.” The log-ratio transformation matrix **G** has dimensions *N* − 1 × *N* and, therefore, is not uniquely invertible (hence the quotation marks). However, if we assume that **V** is sparse then it is possible to uniquely solve for **V** in terms of the inferred $\tilde{V}$. In the context of our ecological model, this assumption means that any individual consumer species is unlikely to be able to utilize every effective resource. The algorithm for sparse inversion of the log-ratio transformation (Materials and Methods and S1 Text) was applied to the component matrices obtained from both PCA and CoCA before performing any species comparisons based on the inferred resource utilizations.

Log-ratio transformations (S1 Text) ensure that Euclidean distances in the transformed space can be used to estimate beta diversity—though, only the isometric log-ratio transform ensures that these distances coincide with the Aitchison metric for compositional data (S1 Fig). It is, of course, possible to choose from many different distance metrics, such as Bray-Curtis or UniFrac [25, 26]. Using other metrics, an appropriate dimensionality reduction is Principal Coordinates Analysis (PCoA), or classical multidimensional scaling [27], rather than PCA. It is also possible to develop an analogous CoCA for these alternative distance metrics, but the theoretical interpretation as a consumer-resource system derived from the maximum diversity hypothesis would no longer apply.

### Analysis

**Fig 2:**
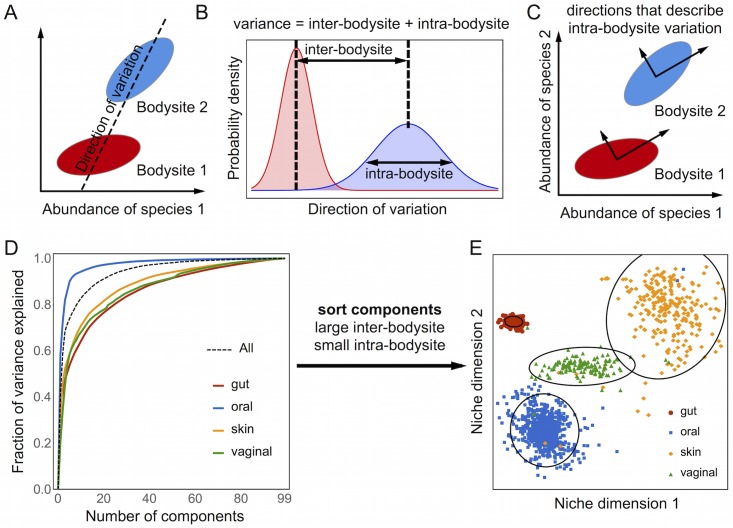
Describing intra-bodysite variability with common components analysis. A) Covariation of the relative abundances along any direction captures both inter-bodysite differences and intra-bodysite variability. B) The law of total variance states that the variance along a direction is variance = (inter-bodysite differences)^2^ + intra-bodysite variance. C) Common Components Analysis (CoCA) finds a common set of directions that simultaneously capture intra-bodysite variability in each of the four bodysites. D) Most of the intra-bodysite variability in species composition can be explained with a small number of common components. E) Projecting the samples onto two common components with large inter-bodysite differences and small intra-bodysite variation clearly separates the four body sites.

Although we have stated the maximum diversity hypothesis as a generative model of species composition, we rarely know, and generally cannot measure, all of the effective resources in a community. Therefore, we treat the mathematical model as an inverse problem with the goal of inferring the effective resources from observations of species composition across many individuals and bodysites. The inverse problem can be solved because, by construction, the model imposes that the inferred effective resources correspond to directions with high intra-bodysite variability (Fig 2A–2C). We exploit this feature to developed a technique we called CoCA that infers the characteristics of the species and habitats from observed correlations (S1 Text). Like other techniques for simultaneous matrix diagonalization [19–21, 28], CoCA aims to find a single set of directions that simultaneously explain variation within each of the bodysites. Moreover, CoCA has a theoretical interpretation derived from the maximum diversity hypothesis and properly accounts for the compositional nature of genomic survey data.

Our statistical analysis of the data from the Human Microbiome Project (HMP) centers on three observations:

1. The covariance matrices from the bodysites are simultaneously diagonalizable and, thus, have a common basis.
2. Species that are close together in this common basis are also closely related taxonomically.
3. (Corollary) Distances between species in the common basis can be predicted from the similarities of the species in a small number of metabolic pathways.

The first point is a validation of our mathematical model, whereas points 2 and 3 demonstrate that the results obtained by CoCA have biologically reasonable interpretations. Point 3 is a corollary of point 2; if taxonomy explains species relationships in the common basis then it follows that there will also be a relationship with characteristics that vary by taxonomy (e.g., metabolic pathways). Nevertheless, it is important to check that the pathways that are selected make biological sense. We compare the CoCA results to PCA, as well as to the results of the CoCA algorithm applied to randomized data (S1 Text).

#### The covariance matrices from the bodysites are simultaneously diagonalizable and, thus, have a common basis

We applied CoCA to study the relative abundances of *N* = 100 highly abundant species from the HMP (SI). To validate the mathematical model underlying CoCA, we verified that its assumptions, which are rooted in our hypothesis that compositional variation is driven by habitat fluctuations, are not violated (S1 Text). In general, it is not possible to find single basis that simultaneously diagonalizes multiple covariance matrices. Thus, the CoCA algorithm will fail actually fail if the underlying data are not generated by a set of latent variables reflecting a common biological process underlying intra-bodysite variability. PCA, by contrast, always finds the basis that explains the maximum *total* (rather than intra-bodysite) variation using the smallest number of components, even if the assumptions underlying the generative model (e.g. normality) are violated.

**Fig 3:**
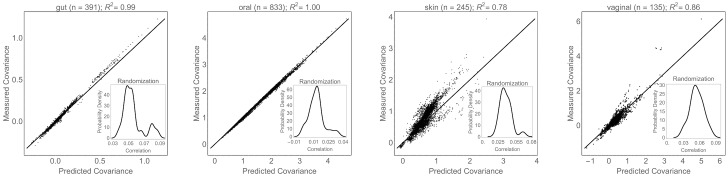
Goodness-of-fit of the common components analysis model. Correlations between the observed covariances (**Σ**_***s***_) and those computed from CoCA ($\tilde{V}\Sigma_{s}{\tilde{V}}^{T}$) in each of the 4 body sites. (Insets) The distribution of correlations from all 20 randomizations, which are roughly an order of magnitude smaller than the observed values.

We tested our model by applying CoCA to both the real covariance matrices constructed from data, and to randomized covariance matrices that do not have a common basis (S1 Text). The top row of Fig 3 shows that CoCA identifies a common basis that explains the observed covariances quite well, with *R*^2^ ≥ 0.78. As expected, the second row of Fig 3 shows that the CoCA algorithm cannot fit the covariance matrices after randomization, with *R*^2^ = 0 in all bodysites. In contrast to PCA, the vectors defining the common basis do not need to be orthogonal. Therefore, it is also necessary to check that variation along these directions is uncorrelated within each bodysite. S2 Fig shows that the variances along each direction agree nearly perfectly with the inferred variances (i.e., *Σ*_*ij*|*s*_), and S3 Fig shows that the correlations between the inferred components are small. Taken together, these results demonstrate that observed covariance matrices are approximately simultaneously diagonalizable, and that the observed performance is not due to chance.

Fig 2D shows that CoCA identifies a few components (i.e., effective resources) that explain most of the intra-bodysite variation in the human microbiota. Even though the CoCA components only capture the directions that explain intra-bodysite variability, they can be ranked by the ratio of how much they vary between bodysites to how much they vary within bodysites. Sorting the CoCA components in this way identifies directions that clearly separate all four bodysites into coherent clusters (Fig 2E). By contrast, the principal components are typically ranked based on their contribution to total variability, which is a mixture of inter- and intra-bodysite variation. Thus, highly ranked principal components may correspond to directions with large intra-bodysite variations, causing them to miss directions with large inter-bodysite differences. As a result, the two largest principal components are unable to separate all four bodysites (S5 and S6 Figs).

#### Species that are close together in this common basis are also closely related taxonomically

**Fig 4:**
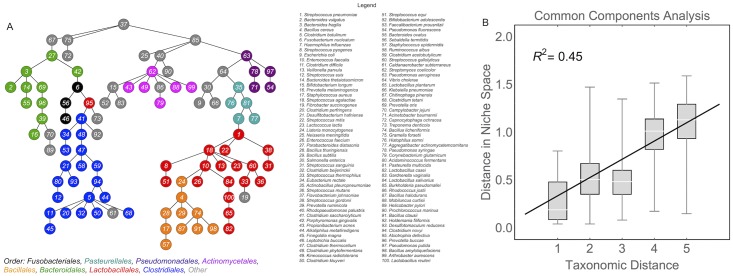
Visualizing the niche space of the human microbiota. Each species can be described as a vector within a niche space that describes the ability of a species to utilize the effective resources, weighted by the average variability of that resource within each of the bodysites. Distances between two species in this niche space were quantified with a metric that measures the angle the two vectors (S1 Text). A) The minimum spanning tree of the niche space connects all of the species so that the average distance between connected species is as small as possible. Thus, two species are connected if they have similar effective resource utilizations. The species are colored according to their taxonomic classification at level ‘order’. Only the top eight orders with the most representative species are colored; species in underrepresented orders are shown in gray. B) The distance between species in the niche space obtained from CoCA is strongly related to species similarity. Here, the taxonomic distance between two species is five minus the number of overlapping taxonomic levels. See SI for discussion of statistical significance.

CoCA describes each species as a vector where, after recovery of the sparse matrix **V** from the inferred $\tilde{V}$, each element of the vector describes the ability of that species to use one of the effective resources. Thus, the angle between two of these vectors describes how similar the two species are in terms of their abilities to use the effective resources. Species that are positively correlated are close together in this ‘niche space’, whereas species that are uncorrelated (or anti-correlated) are far apart. Fig 4A shows all of the species connected into a tree, so that each species is only connected to other species with similar effective resource utilizations. Coloring the tree by taxonomic classification at the level of ‘order’ reveals that the species cluster into taxonomically coherent groups [29]. In fact, the more similar two species are in terms of taxonomy, the closer they are in this niche space (Fig 4B; S4 and S6 Figs). To put it another way, the relative abundances of related species are highly correlated because they have similar resource requirements. This is true even though species that use similar resources are competing with each other. The intuition derived from dynamical models that the abundances of competing species should be anti-correlated simply does not apply when the habitat conditions are highly variable.

#### Distances between species in the common basis can be predicted from the similarities of the species in a small number of metabolic pathways

**Fig 5:**
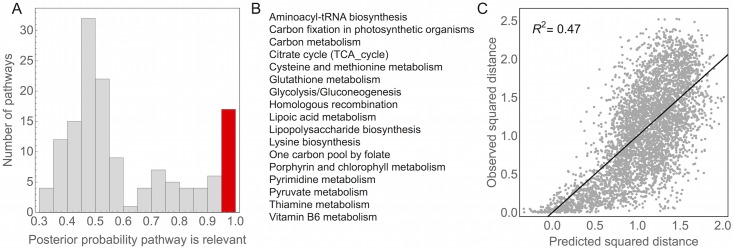
Functional pathways related to inter-species distances in niche space. We performed a linear regression of the squared distances computed from CoCA against the squared distances computed from KEGG functional pathways (S1 Text). Each pathway was assigned a probability that it contributes to the distances between species in niche space (i.e. that the regression coefficient associated with the pathway is nonzero) using a Bayesian model selection algorithm (S1 Text). A) A histogram of the probabilities associated with each of the KEGG pathways. We defined pathways as significantly associated if they had a posterior probability greater than 0.95, and the bar representing the significant pathways is shown in red. B) A list of the relevant functional pathways in alphabetical order. C) The regression using just these relevant pathways has a correlation coefficient of *R*^2^ = 0.47.

So far, we have described the components derived from CoCA as abstract resources that represent unknown abiotic and biotic factors in the habitat. To check that these effective resources correspond to biologically meaningful functions, we regressed the distances between species in the CoCA derived niche space against inter-species distances computed from KEGG functional pathways (S1 Text) [30, 31]. As we have focused on the taxonomic description of communities, we compiled functional information from taxonomic classifications to maintain the same level of granularity (S1 Text). A similar analysis could be performed by mapping CoCA effective resources to functional categories directly from whole genome sequencing data, or from functional information estimated using PICRUSt or Tax4Fun [32, 33] for 16S community surveys. We used a statistical technique called the Bayesian Ising Approximation to assign a posterior probability to each KEGG pathway [34, 35] (Materials and Methods and S1 Text). The posterior probability is a measure of degree of belief; it quantifies how relevant each KEGG pathway is for computing the similarity between species derived from CoCA. A histogram of the posterior probabilities is shown in Fig 5A (also S7 and S8 Figs). We designated pathways as relevant if they had a posterior probability greater than 0.95. The 17 pathways reaching this threshold for relevance are listed in alphabetical order in Fig 5B. Taken together, these relevant pathways explain roughly half of the variation in the distances between species in the CoCA derived niche space (Fig 5C). Thus, the percentage of variance explained by the pathways is roughly equivalent to the variance explained by taxonomy, as one would expect if the two analyses pick up on the same underlying relationship. The relevant pathways are primarily associated with carbon metabolism or maintenance of the cell wall. Thus, CoCA highlights the ecological separation between aerobic and anaerobic species and between gram positive and gram negative bacteria, pointing to the importance of both metabolic processes and host-microbiome interactions for structuring the human microbiota.

### Conclusion

Previous studies have revealed that bacteria exhibit tremendous genomic and functional diversity due, in part, to high rates of horizontal gene transfer (HGT) [36]. As a result, the ability of sequence-based or taxonomic classification of bacteria to capture ecological relationships has been called into question [37–40]. Nevertheless, we found that genetically related species respond to fluctuating habitat conditions in the same way, implying that they occupy similar ecological niches. Thus, current taxonomic groupings of bacteria are largely sufficient for explaining cross-sectional correlations in relative species abundances over the healthy human population. This result is not at odds with high rates of HGT; it simply implies ecologically derived constraints on evolution.

We introduced CoCA, a theory-driven data analysis technique that can be applied to any cross-sectional study with labeled metadata, including studies with populations corresponding to healthy and unhealthy individuals. Although the effective resources identified by CoCA are derived entirely from data on relative species abundances across a population, they reflect indirect ecological relationships between species that are mediated through resources and form the basis of a common resource sharing niche space of the human microbiota. Future analyses of larger, and more diverse, datasets will further elucidate the relationship between this underlying niche space and the functional properties of the organisms in the microbiota. Given that CoCA identifies features that separate the bodysites with high fidelity, we believe that it is a useful technique for identifying microbiota based biomarkers that discriminate between host phenotypes. Extending our results to include data from unhealthy subjects will be an important avenue for future work.

A recent paper by Bashan et al [41] developed an approach to analyzing microbial dynamics based on a Dissimilarity-Overlap Curve. They found that communities with a high overlap in the species that were present also have a low dissimilarity in their relative abundance profiles. They argue that this relationship is evidence of “universality” where interspecies interactions are essentially the same across a population of human subjects. Our model is also based on the assumption that the underlying drivers of variation in the microbiota are the same across subjects and across bodysites, and it is only the relative importance of these factors that leads to differences between groups. However, we focused only on variation in the relative abundances of highly abundant species that are present across all four major bodysites in the Human Microbiome Project rather than variation in species assemblages.

The successful application of CoCA to HMP data from four different body sites implies that the processes that shape the variation in species abundances are shared between bodysites, and only changes in the specific contribution of various effective resources differentiate bodysites. CoCA uses simultaneous diagonalization to identify processes that are shared between communities. Covariance matrices from communities without shared drivers of variation cannot be simultaneously diagonalized, as we showed with randomized data. Consequently, we would expect that CoCA would fail on datasets from clearly different ecological environments (e.g., hot springs compared to body sites). In this case, failure is not a bad thing: it can be easily diagnosed from the poor agreement between the predicted and observed covariances and it provides an ecologically meaningful result be ruling out the hypothesis that the environments have shared drivers of variation.

CoCA does not explain 100% of the variation in the HMP data, nor do taxonomic relationships explain 100% of the variation in the inferred resource utilizations. The additional variation is likely do to other types of interactions between species in the human microbiota that cannot be captured using effective resources that are shared across bodysites. Moreover, effective resources are only defined by a statistical model and, therefore, do not have obvious relationships to measurable environmental variables. Here, we attempted to explain the inferred effective resources in terms of metabolic processes inferred through KEGG pathways but it is likely that other factors, such as resilience to temperature or pH ranges, contribute to the effective resources in ways that our analysis with KEGG pathways could not uncover.

Our study also has other limitations that should be addressed in future work. We have based our analyses on relative species abundances derived from OTUs constructed using data from the HMP. These data are likely to be noisy, but the degree of uncertainty is difficult to quantify. Moreover, the use of OTUs defined by 97% sequence identity, and subsequent reduction of the communities to 100 highly abundant species, leads to a coarse grained representation that may smooth out relevant features. It will be important to revisit our analyses on additional datasets, and with additional tools for generating highly accurate pictures of community composition. On the theoretical side, it will be important to examine the validity of the maximum diversity hypothesis across communities with different properties.

## Methods

### Data collection

We analyzed data from the Human Microbiome Project (HMP) on person-to-person variability in relative species abundances in four bodysites (gut, oral cavity, vagina, and skin; Fig 1A) [3–5, 12]. The species-level relative abundances derived from the HMP whole genome sequencing data were obtained from MG-RAST (Project 385) through the MR-RAST API [42]. Only the processed data as provided on the MG-RAST server were extracted. Thus, these species counts were constructed using the default MG-RAST pipeline [43]. Briefly, this pipeline identifies putative rRNA fragments and clusters them at 97% identity to define operational taxonomic units, which are assigned species labels using a search against the M5rna database [44]. We eliminated any lowly abundant species and selected for further study 100 species (Fig 3A) that were highly abundant across all bodysites, as described in S1 Text. The final dataset (consisting of the counts of the 100 selected species in each of the samples) is available in the Supporting Information (S1 Code) and at https://sites.google.com/site/charleskennethfisher/home/programs-and-data along with the source code.

### Log-ratio transformations and CoCA

Log-ratio transformations are obtained using ***y*** = **G** log ***x***, where ***x*** is an *N* dimensional vector of relative abundances, **G** is an *N* − 1 × *N* matrix with **G1** = **0**, and ***y*** is an *N* − 1 dimensional vector of transformed relative abundances. We use a **G** that implements an additive log-ratio (or ALR) transform, but the choice of **G** is not critical for our analyses and some other possible choices are discussed further in the S1 Text. Applying a log-ratio transformation to Eq 2 gives $y=GV\lambda =\tilde{V}\lambda$. Here, **V** is an *N* × *N* − 1 dimensional matrix whereas $\tilde{V}=GV$ is an *N* − 1 × *N* − 1 dimensional matrix. Once again, the use of relative abundances shows up as a loss of information. The matrix **V** that contains the information about all *N* species that we would like to obtain can only be recovered from $\tilde{V}$ using some assumptions.

The use of compositional transformations with CoCA requires an extra step to recover the *N* × *N* − 1 dimensional matrix **V** from the *N* − 1 × *N* − 1 dimensional matrix $\tilde{V}=GV$. Unfortunately, the matrix **G** is not invertible. But, if we assume that **V** is sparse then it is possible to determine **V** from $\tilde{V}$. In the context of the model, this assumption means that any individual consumer species is unlikely to be able to utilize every effective resource. To recover **V** from $\tilde{V}$, we solve the problem:

$$\min{\left||V|\right|}_{1}\;\text{subject}\;\text{to}\;GV=\tilde{V}$$ (5)

where ||**V**||_1_ = ∑_*iμ*_ |*V*_*iμ*_|. Using the ALR transform, all of the solutions to this problem are all of the form $V_{i\mu }=z_{\mu }+{\tilde{V}}_{(i-1),\mu }\left(1-\delta_{i1}\right)$ for *i* = 1, …, *N*, where *z*_*μ*_ can, in principle, take on any real value. Because we want the solution with a minimum *L*_1_ norm, it is sufficient to test *z*_*μ*_ = 0 and $z_{\mu }\in {\left\{-{\tilde{V}}_{i,\mu }\right\}}_{i=1}^{N-1}$ (the only sparse solutions) and to choose the one with minimum norm. This is a tractable search over *N*(*N* − 1) possibilities in the worst case and can be done easily for reasonable system sizes.

### Pathway selection with the Bayesian Ising Approximation

Each row of the matrix $V=G\tilde{V}$ (here, **G** is a matrix that arises from the log-ratio transform—see details) describes how one of the species responds to changes in the latent variables. Thus, the *i*^*th*^ row of **V** is a mathematical representation of species *i*. The distance between species *i* and *j* in the inferred basis can be calculated by computing the distance between the *i*^*th*^ and *j*^*th*^ rows of **V** with each column (i.e., latent variable) weighted by its variance (S1 Text).

Distances between species computed from the common components were regressed against the distances computed from KEGG pathways (S1 Text). To select relevant pathways, we compute posterior probabilities for each regression coefficient to be non-zero using the Bayesian Ising Approximation (BIA) [34, 35]. The BIA approximates the posterior distribution of a vector indicator variables with *s*_*i*_ = +1 if pathway *i* relevant and *s*_*i*_ = − 1 if pathway *i* is not relevant. The posterior distribution is approximately an Ising model described by:

$$\log\;P_{\lambda }\left(s|y\right)≃\frac{n^{2}}{4\lambda }\left(\sum_{i}h_{i}\left(\lambda \right)s_{i}+\frac{1}{2}\sum_{i,j;i\neq j}J_{ij}\left(\lambda \right)s_{i}s_{j}\right)$$ (6)

where the external fields (*h*_*i*_) and couplings (*J*_*ij*_) are defined as:

$$h_{i}\left(\lambda \right)=r^{2}\left(y,x_{i}\right)-\frac{1}{n}+\sum_{j}J_{ij}\left(\lambda \right)$$ (7)

$$J_{ij}\left(\lambda \right)=\lambda^{-1}r^{2}\left(x_{i},x_{j}\right)-\frac{n}{\lambda }\left(r\left(x_{i},x_{j}\right)r\left(y,x_{i}\right)r\left(y,x_{j}\right)-\frac{1}{2}r^{2}\left(y,x_{i}\right)r^{2}\left(y,x_{j}\right)\right)$$ (8)

and λ is the inverse variance of the prior distribution. Here, *r*(*z*_1_, *z*_2_) is the Pearson correlation coefficient between variables *z*_1_ and *z*_2_. The BIA approximation is based on a series expansion that is valid as long as:

$$\lambda \geq \lambda^{*}=n\left(1+pr\right).$$ (9)

where $r=\sqrt{p^{-1}{\left(p-1\right)}^{-1}\sum_{i\neq j}r^{2}\left(X_{i},X_{j}\right)}$ is the root mean square correlation between features.

To perform feature selection, we are interested in computing marginal probabilities *P*_λ_(*s*_*j*_ = 1|**y**) ≃ (1 + *m*_*j*_(λ))/2, where we have defined the magnetizations *m*_*j*_(λ) = 〈*s*_*j*_〉. While there are many techniques for calculating the magnetizations of an Ising model, we focus on the mean field approximation which leads to a self-consistent equation:

$$m_{i}\left(\lambda \right)=\tanh\left[\frac{n^{2}}{4\lambda }\left(h_{i}\left(\lambda \right)+\frac{1}{2}\sum_{j\neq i}J_{ij}\left(\lambda \right)m_{j}\left(\lambda \right)\right)\right]$$ (10)

This mean field approximation provides a computationally efficient tool that approximates Bayesian feature selection for linear regression.

## Supporting information

S1 Fig Comparison of the additive logratio (ALR) transform with the Aitchison distance. A) The Aitchison distance is a metric for relative abundance data based on log-ratios. The ALR transform is not isometric (meaning, it does not preserve distances exactly) but the distances computed with the ALR transformed relative abundances are highly correlated the Aitchison distance (R = 0.87). b) The first two principal coordinates computed using the Aitchison distance. c) The first two principal coordinates computed using the ALR transformed relative abundances (reproduced in S7E Fig).
(TIFF)

S2 Fig Fitting the common components analysis model. Plot of the CoCA objective function during gradient descent using the true covariance matrices (red, dashed line) and 20 randomized covariances matrices (black lines). The error bars on the final value of the objective function with the randomized matrices represent ± 6 standard deviations.
(TIFF)

S3 Fig Comparing the variance in λ to Σ_*s*_. (Top row) Correlations between diagonal elements of **Σ_*s*_** and the variances computed from the inferred **λ**’s. (Middle row) Correlations between diagonal elements of **Σ_*s*_** and the variances computed from the inferred **λ**’s for the best of the 20 randomizations. (Bottom row) The distribution of correlations from all 20 randomizations.
(TIFF)

S4 Fig Intra-body site correlations between common components. Histograms of the correlations between λ_*μ*_ and λ_*ν*_, conditioned on body site, computed from observed covariance matrices (top row) and randomized covariance matrices (bottom row). These plots show that the niche availabilities obtained from the observed data are approximately uncorrelated, whereas those inferred from randomized covariance matrices are not.
(TIFF)

S5 Fig Correlation between taxonomic and ecological distances. Histogram of the correlation between the taxonomic distance and ecological distances computed from randomized covariance matrices. The correlation obtained with the observed data is shown as a dotted red line. The true correlation lies far outside the distribution obtained from randomization.
(TIFF)

S6 Fig Schematic comparison of CoCA and PCA. Each species corresponds to a point in a high dimensional niche space. Fluctuations in the availabilities of the niches from person-to-person cause fluctuations in the relative abundances of the species. If the distribution of niche availabilities does not depend on the bodysite (e.g. gut, skin, etc) then the log-ratio transformed abundances are Gaussian distributed, and the structure of niche space can be inferred using Principal Components Analysis (PCA) by finding the set of axes with the largest variation. If the distribution of the niche availabilities does depend on the bodysite, however, then the log-ratio transformed abundances are drawn from mixture of Gaussians and maximum likelihood fitting of the model identifies a common set of axes, or common components, that approximately diagonalize the covariance matrices in each of the bodysites.
(TIFF)

S7 Fig Comparison of CoCA and PCA on the HMP data. A) Percentage of variance explained in each bodysite as function of the number of common components. B) Projecting onto two common components with large inter-bodysite differences and small intra-bodysite variation separates the bodysites into coherent clusters. C) Distances between species computed from CoCA are strongly correlated with taxonomy. Note that parts A-C are reproduced from the Main Text to facilitate comparison with PCA. D) Percentage of variance explained in each bodysite as function of the number of principal components. E) Projecting onto the two largest principal components fails to separate the bodysites into coherent clusters. F) Distances between species computed from PCA are only weakly correlated with taxonomy.
(TIFF)

S8 Fig Feature selection path of the Bayesian Ising Approximation. Posterior probability that each figure (i.e., KEGG pathway) is relevant for computing the ecological distance between species as a function of the variance of the prior distribution (i.e., the inverse of the regularization parameter). The pathways with a posterior probability greater than 0.95 when the inverse regularization parameter is one (i.e, λ*/λ = 1) are shown in red.
(TIFF)

S9 Fig Comparison of BIA to Monte Carlo simulations. Posterior probabilities estimated using the BIA compared to those computed with Monte Carlo simulations for λ = λ*. Four pathways (shown) reach a posterior probability of 0.95 for Monte Carlo, but not for BIA. All pathways that reached the 0.95 threshold for relevance with the BIA also reached the relevance threshold with Monte Carlo.
(TIFF)

S1 Text Extended sections on theory and materials and methods. (PDF)

S1 Code Zip archive containing the CoCA code and the processed HMP data. (ZIP)
