# Supplementary figures and images for: Variable habitat conditions drive species covariation in the human microbiota
Source: PLoS Comput Biol. 2017 Apr 27;13(4):e1005435. doi: 10.1371/journal.pcbi.1005435 (PMC5407567; doi:10.1371/journal.pcbi.1005435)

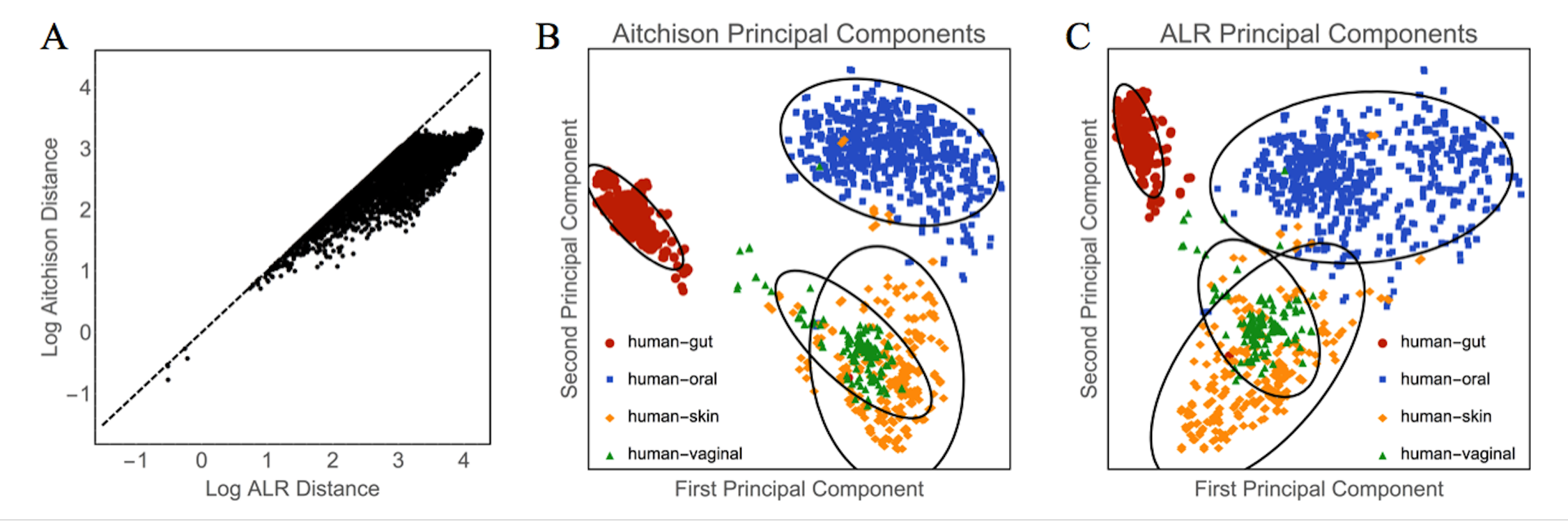

Supplement: S1 Fig — A) The Aitchison distance is a metric for relative abundance data based on log-ratios. The ALR transform is not isometric (meaning, it does not preserve distances exactly) but the distances computed with the ALR transformed relative abundances are highly correlated the Aitchison distance (R = 0.87). b) The first two principal coordinates computed using the Aitchison distance. c) The first two principal coordinates computed using the ALR transformed relative abundances (reproduced in S7E Fig). (TIFF) [file pcbi.1005435.s001.tiff]

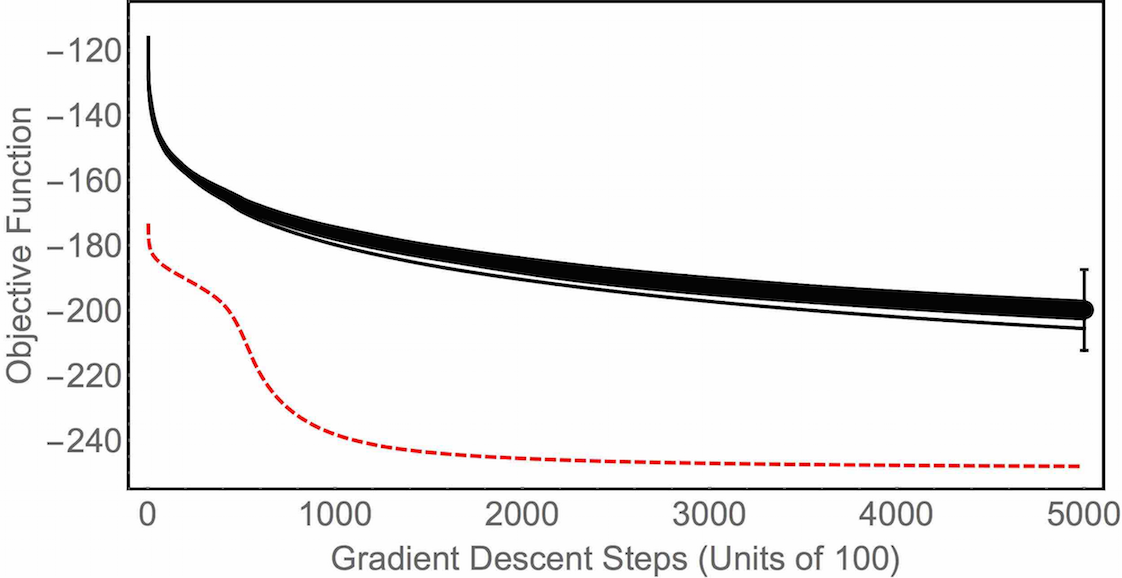

Supplement: S2 Fig — Plot of the CoCA objective function during gradient descent using the true covariance matrices (red, dashed line) and 20 randomized covariances matrices (black lines). The error bars on the final value of the objective function with the randomized matrices represent ± 6 standard deviations. (TIFF) [file pcbi.1005435.s002.tiff]

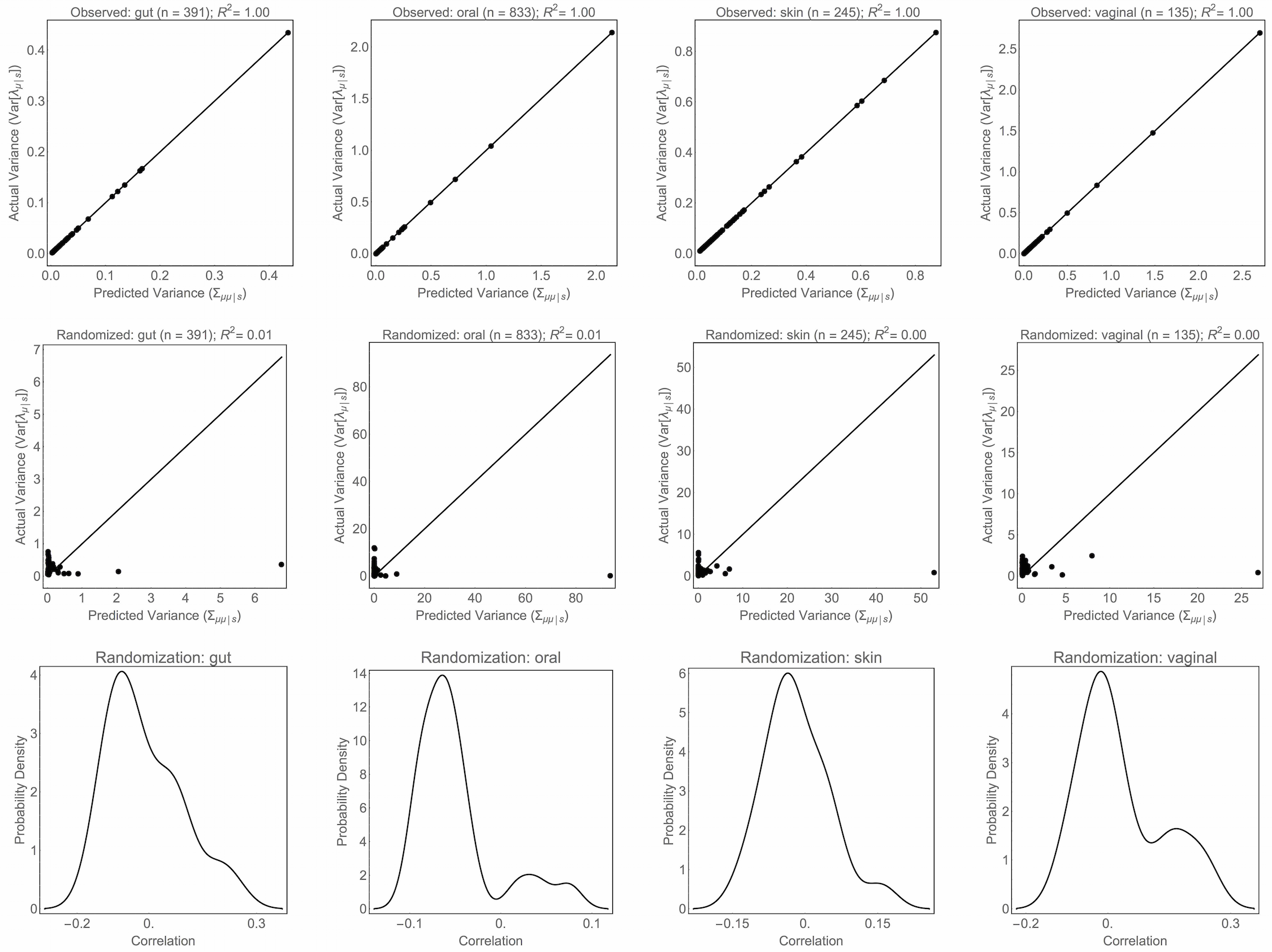

Supplement: S3 Fig — (Top row) Correlations between diagonal elements of Σs and the variances computed from the inferred λ’s. (Middle row) Correlations between diagonal elements of Σs and the variances computed from the inferred λ’s for the best of the 20 randomizations. (Bottom row) The distribution of correlations from all 20 randomizations. (TIFF) [file pcbi.1005435.s003.tiff]

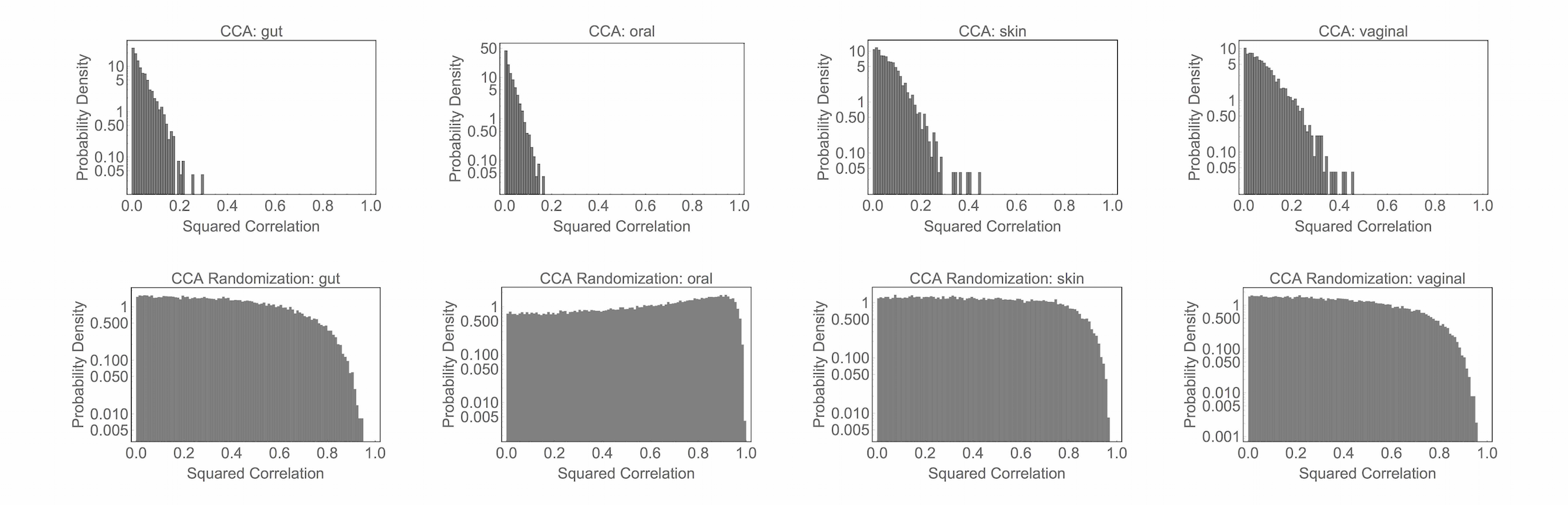

Supplement: S4 Fig — Histograms of the correlations between λμ and λν, conditioned on body site, computed from observed covariance matrices (top row) and randomized covariance matrices (bottom row). These plots show that the niche availabilities obtained from the observed data are approximately uncorrelated, whereas those inferred from randomized covariance matrices are not. (TIFF) [file pcbi.1005435.s004.tiff]

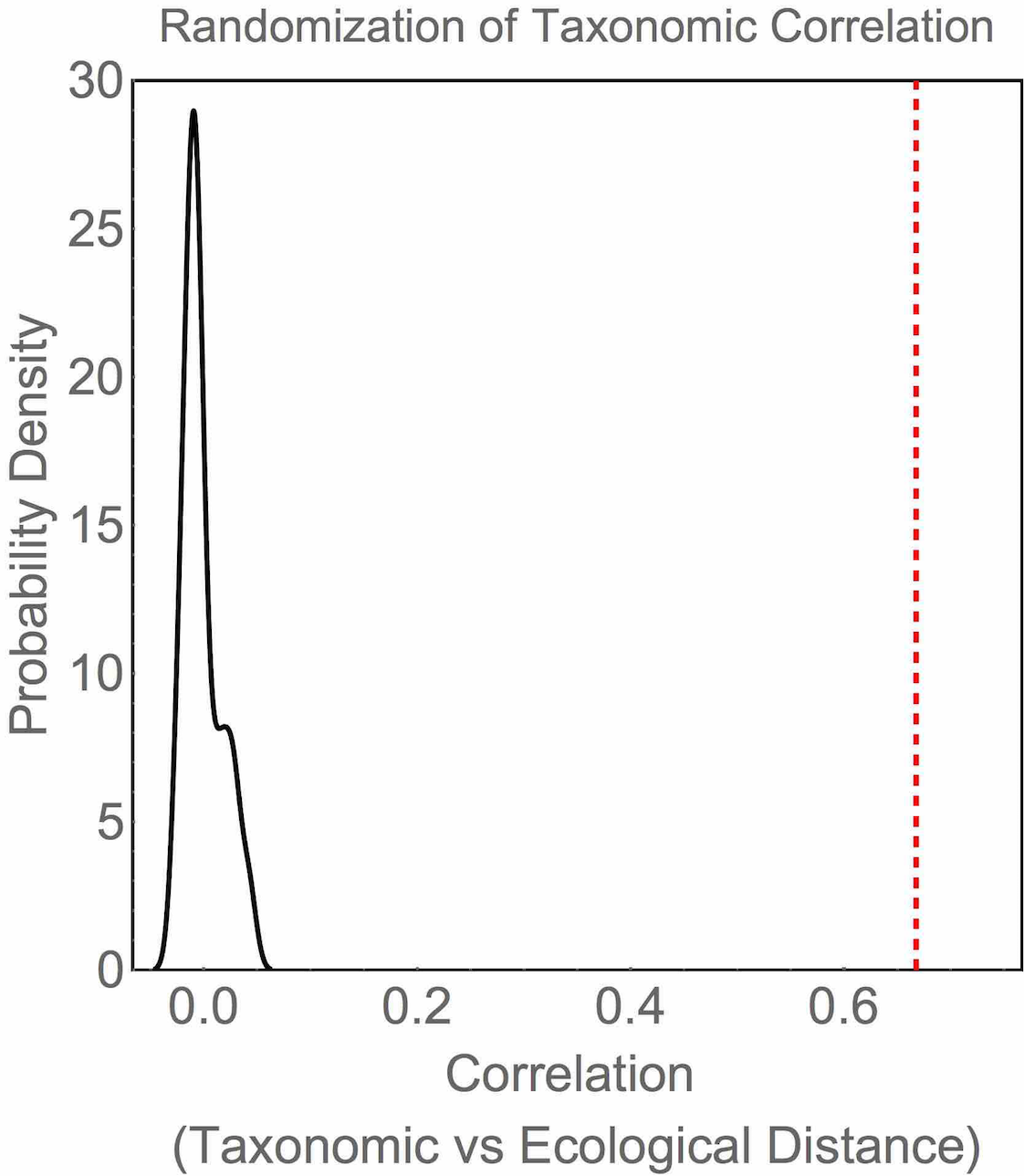

Supplement: S5 Fig — Histogram of the correlation between the taxonomic distance and ecological distances computed from randomized covariance matrices. The correlation obtained with the observed data is shown as a dotted red line. The true correlation lies far outside the distribution obtained from randomization. (TIFF) [file pcbi.1005435.s005.tiff]

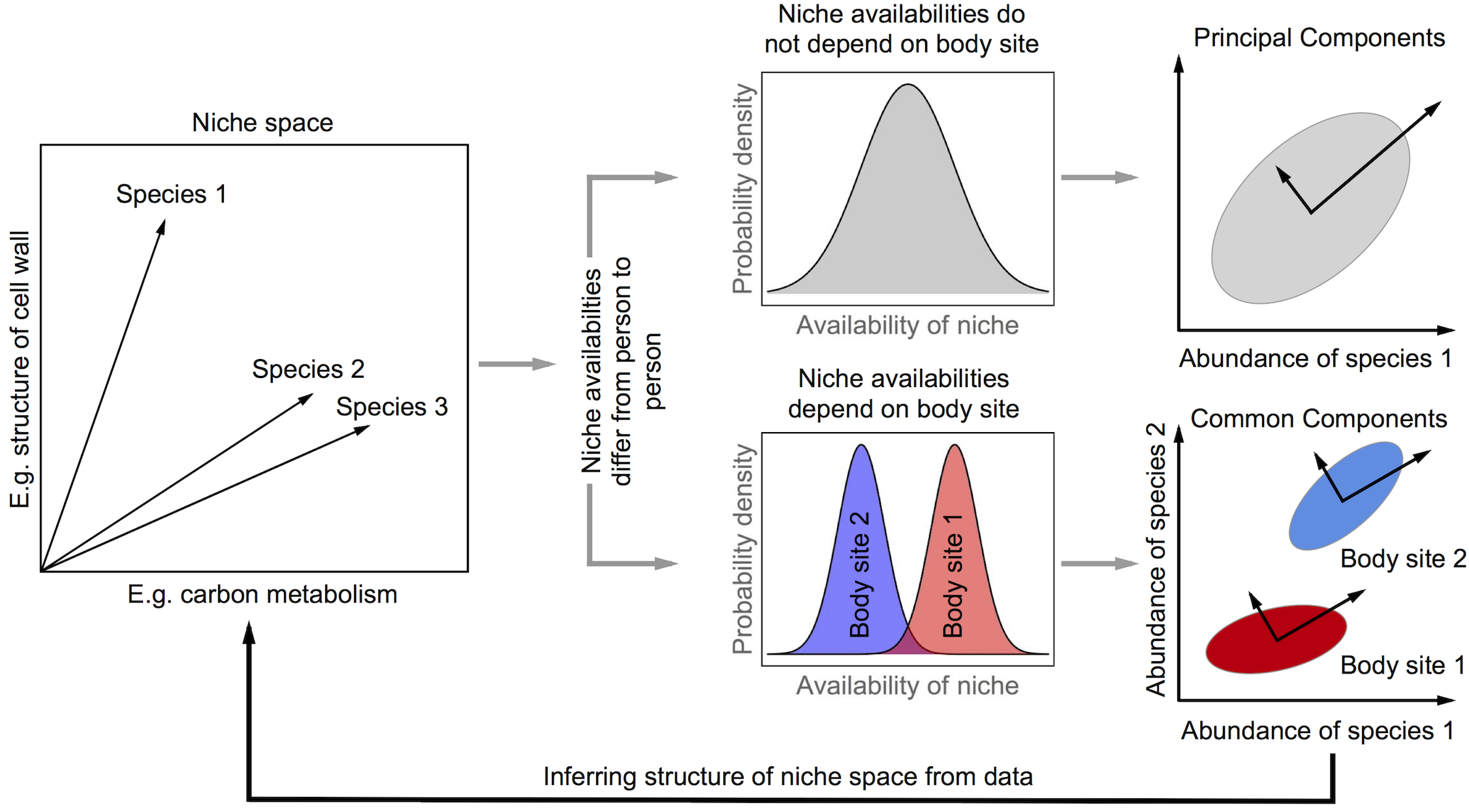

Supplement: S6 Fig — Each species corresponds to a point in a high dimensional niche space. Fluctuations in the availabilities of the niches from person-to-person cause fluctuations in the relative abundances of the species. If the distribution of niche availabilities does not depend on the bodysite (e.g. gut, skin, etc) then the log-ratio transformed abundances are Gaussian distributed, and the structure of niche space can be inferred using Principal Components Analysis (PCA) by finding the set of axes with the largest variation. If the distribution of the niche availabilities does depend on the bodysite, however, then the log-ratio transformed abundances are drawn from mixture of Gaussians and maximum likelihood fitting of the model identifies a common set of axes, or common components, that approximately diagonalize the covariance matrices in each of the bodysites. (TIFF) [file pcbi.1005435.s006.tiff]

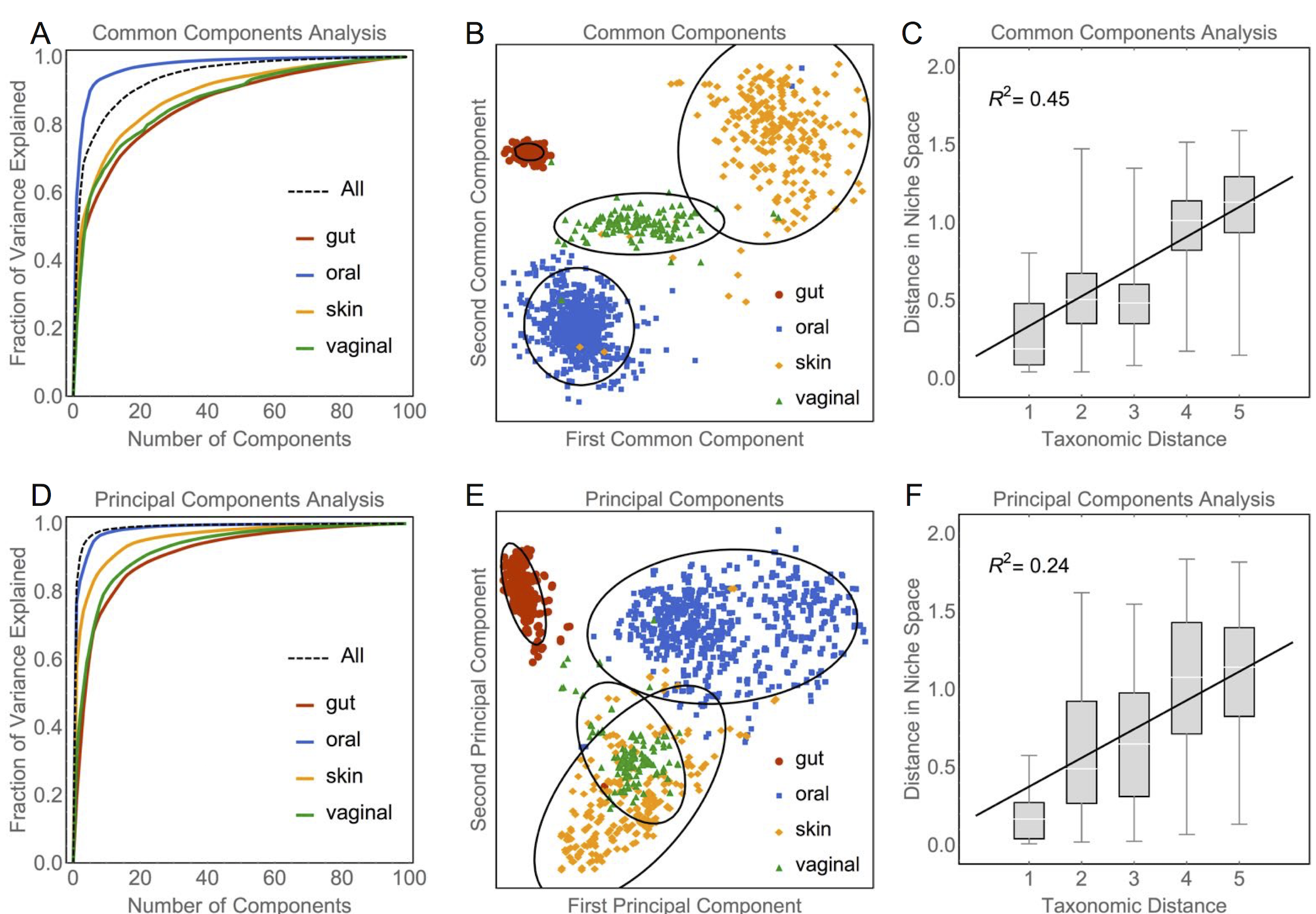

Supplement: S7 Fig — A) Percentage of variance explained in each bodysite as function of the number of common components. B) Projecting onto two common components with large inter-bodysite differences and small intra-bodysite variation separates the bodysites into coherent clusters. C) Distances between species computed from CoCA are strongly correlated with taxonomy. Note that parts A-C are reproduced from the Main Text to facilitate comparison with PCA. D) Percentage of variance explained in each bodysite as function of the number of principal components. E) Projecting onto the two largest principal components fails to separate the bodysites into coherent clusters. F) Distances between species computed from PCA are only weakly correlated with taxonomy. (TIFF) [file pcbi.1005435.s007.tiff]

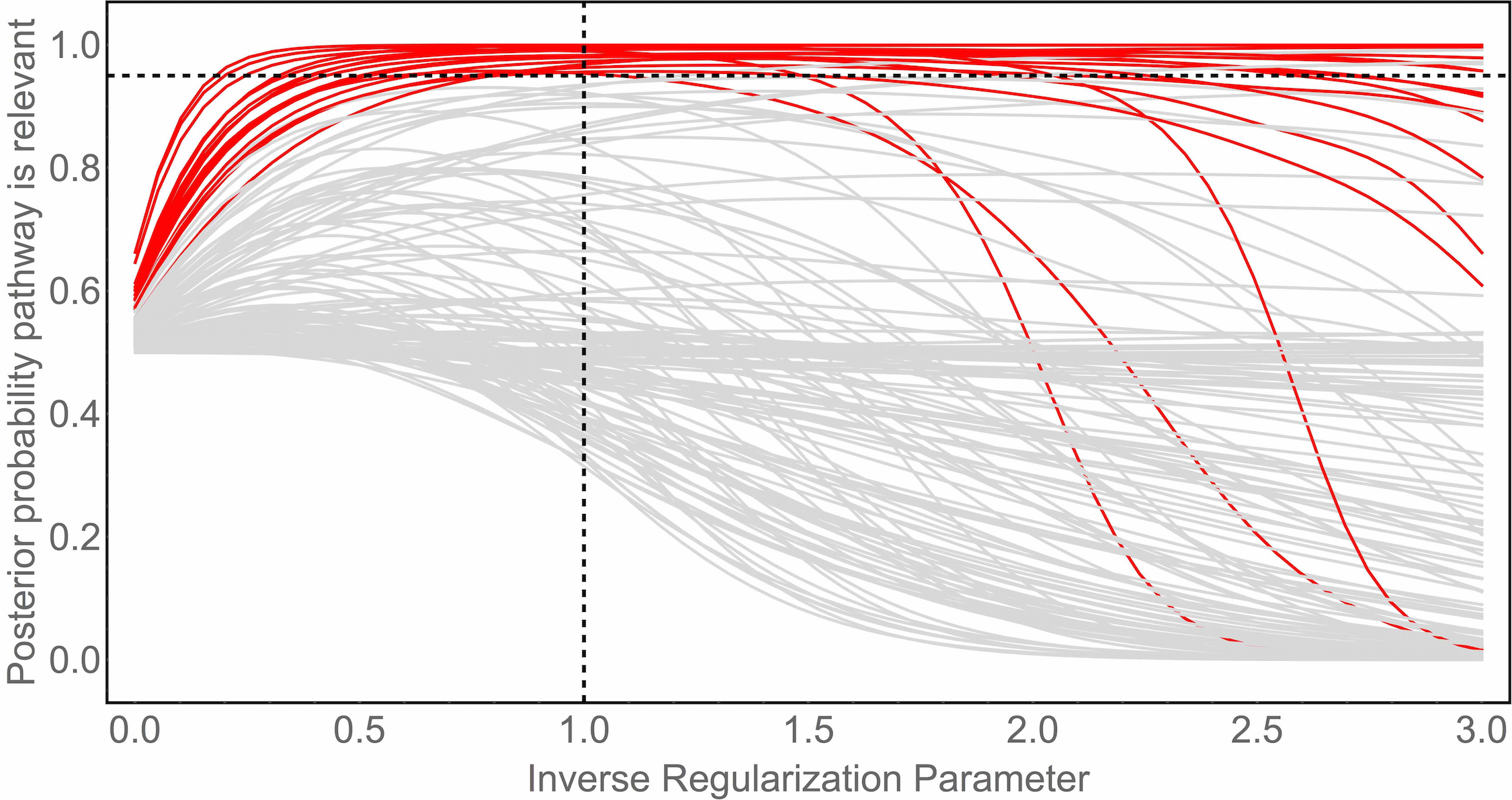

Supplement: S8 Fig — Posterior probability that each figure (i.e., KEGG pathway) is relevant for computing the ecological distance between species as a function of the variance of the prior distribution (i.e., the inverse of the regularization parameter). The pathways with a posterior probability greater than 0.95 when the inverse regularization parameter is one (i.e, λ*/λ = 1) are shown in red. (TIFF) [file pcbi.1005435.s008.tiff]

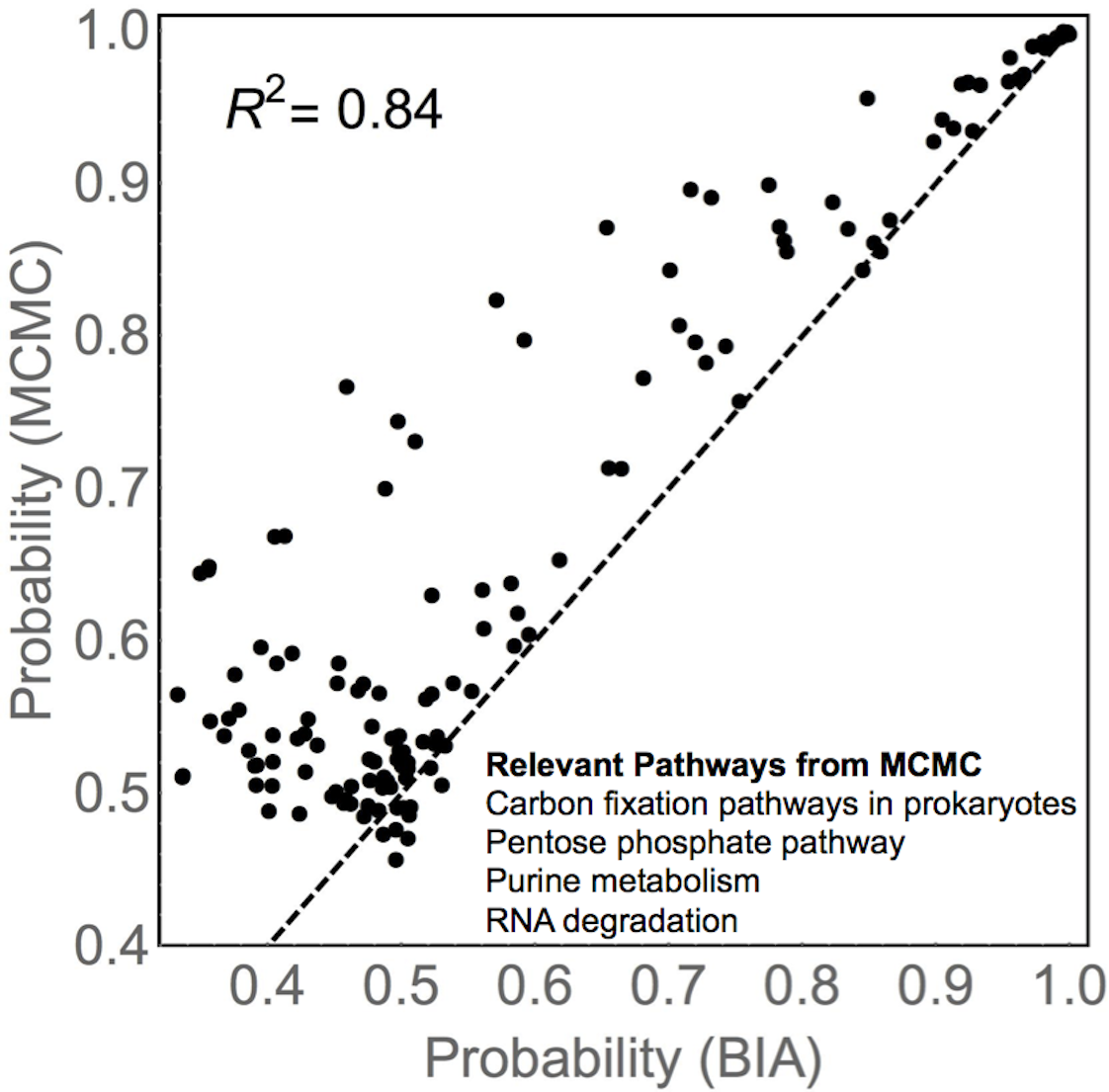

Supplement: S9 Fig — Posterior probabilities estimated using the BIA compared to those computed with Monte Carlo simulations for λ = λ*. Four pathways (shown) reach a posterior probability of 0.95 for Monte Carlo, but not for BIA. All pathways that reached the 0.95 threshold for relevance with the BIA also reached the relevance threshold with Monte Carlo. (TIFF) [file pcbi.1005435.s009.tiff]
